# Learning to Reason on Tree Structures for Knowledge-Based Visual Question Answering

**DOI:** 10.3390/s22041575

**Published:** 2022-02-17

**Authors:** Qifeng Li, Xinyi Tang, Yi Jian

**Affiliations:** 1 Shanghai Institute of Technical Physics of the Chinese Academy of Sciences, Shanghai 200083, China; gq227@mail.sitp.ac.cn (X.T.); jianyi@mail.sitp.ac.cn (Y.J.); 2 School of Electronic, Electrical and Communication Engineering, University of Chinese Academy of Sciences, Beijing 100049, China; 3 Key Laboratory of Infrared System Detection and Imaging Technology, Chinese Academy of Sciences, Shanghai 200083, China

**Keywords:** tree structure, knowledge base, compositional reasoning, neural module network, attention mechanism

## Abstract

Collaborative reasoning for knowledge-based visual question answering is challenging but vital and efficient in understanding the features of the images and questions. While previous methods jointly fuse all kinds of features by attention mechanism or use handcrafted rules to generate a layout for performing compositional reasoning, which lacks the process of visual reasoning and introduces a large number of parameters for predicting the correct answer. For conducting visual reasoning on all kinds of image–question pairs, in this paper, we propose a novel reasoning model of a question-guided tree structure with a knowledge base (QGTSKB) for addressing these problems. In addition, our model consists of four neural module networks: the attention model that locates attended regions based on the image features and question embeddings by attention mechanism, the gated reasoning model that forgets and updates the fused features, the fusion reasoning model that mines high-level semantics of the attended visual features and knowledge base and knowledge-based fact model that makes up for the lack of visual and textual information with external knowledge. Therefore, our model performs visual analysis and reasoning based on tree structures, knowledge base and four neural module networks. Experimental results show that our model achieves superior performance over existing methods on the VQA v2.0 and CLVER dataset, and visual reasoning experiments prove the interpretability of the model.

## 1. Introduction

Visual question answering (VQA) is an intersecting field of computer vision and natural language processing, which has just been proposed in recent years. The meaning of visual question answering is to fully understand the features of images and texts and make the appropriate decision. In the field of visual question answering, visual reasoning is a very difficult but extremely important problem to be solved. However, most of the existing methods [1,2,3,4,5] are similar to black box operations. For example, the image features extracted by convolution neural network (CNN) are fused with the encoded text features, and then the fused features are fed to the artificial neural network. Therefore, these methods turn the visual question answering into a multi-label classification task. The drawback is that these methods lack the ability of logical analysis and reasoning, and fail to explore why such results are obtained. Furthermore, there are some models [6] dedicated to the study of VQA dataset bias, which is only effective for partial datasets, and the models fail to be widely used. Recently, some work [7,8,9,10,11] has not been satisfied with improving the accuracy of the model, and began to analyze the structure of the image and the question. According to the structural characteristics of the question, the task is divided into several subtasks.

The layout generated by these subtasks is considered as a reasoning map for predicting correct answers. However, the disadvantage of these manually pre-defined subtasks is that the questions and images provide very few features, and thus they are short of some common sense knowledge relationship to fully understand the features in the process of reasoning.

**Figure 1:**
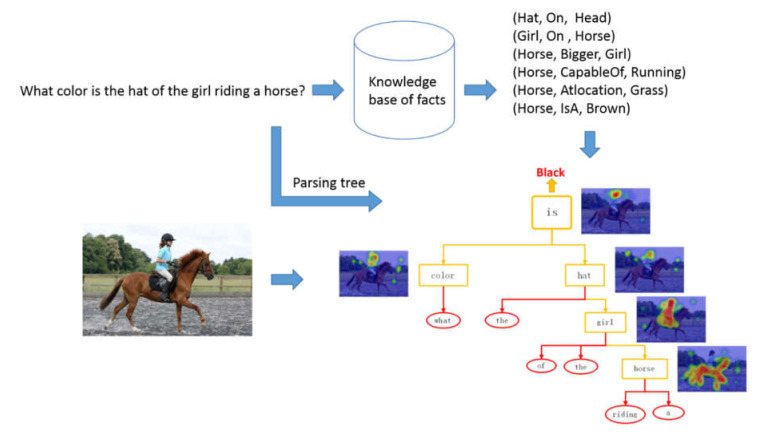
An example of our question-guided tree structure with knowledge base model that from bottom to up performs reasoning over question-guided parsing tree nodes with the help of knowledge-based relation. Given the word nodes, the attention maps are generated using modular networks for explicit visual reasoning.

In this paper, we propose a reasoning model of a question-guided tree structure with a knowledge base (QGTSKB) for addressing these problems. The inherent textual structure of the question is parsed into a tree structure. As shown in Figure 1, our network makes full use of the dynamics of the tree structure and the adaptability of different tasks of the modular network structure, which are utilized to generate a modular reasoning layout. Each word in the question is mapped into the knowledge base, and then the corresponding supporting facts are selected by keyword matching. The task-related and content-complementary facts are essential for building the relationship between images and questions for understanding and reasoning.

In order to solve complex reasoning tasks comprehensively and effectively, we propose four modular networks with different functions, which are the attention module, gated reasoning module, fusion reasoning module and knowledge-based relation module. The attention model not only uses the word encodings of the current tree node, but also fuses the attention map of the child node with the relationship between words from the knowledge base to extract local visual evidence for explicit reasoning. The gated reasoning model is inspired by Gated Recurrent Neural Networks. The advantage of the gated reasoning model is that the information flow can be modulated according to different tasks without a separate memory cell, which can reduce the memory consumption during calculation. Since the gating reasoning model has the function of forgetting and updating, it can delete and update the features passed by the child nodes according to the features of the current node. The fusion model combines the attention features of the current node and the features of the child nodes, which uses bilinear fusion to increase the variability of the fused features so as to enhance the flexibility of the model. The combination of external knowledge and visual information helps humans answer various questions. Therefore, the knowledge base in natural language processing has been gradually paid more attention.

The knowledge-based facts are extracted from the three commonly used knowledge base datasets which are DBPedia [12], WebChild [13] and ConceptNet [14]. The knowledge base model fuses knowledge-based facts and the output representations of two reasoning models, which can make up for the effective information beyond the image and question features.

We conduct extensive experiments to prove that our model has superior performance on CLVER and VQA2.0 public dataset. In addition, the attention map of each node is considered as a qualitative experimental result in the process of explicit visual reasoning, which further shows that our model is interpretable and has strong adaptability to different tasks.

The main contributions are summarized as follows:

1. We propose an interpretable and practical VQA model which unifies the visual, textual, knowledge-based factual representations from different modalities. Given the question-guided parsing tree structure, it performs remarkable reasoning based on the attention mechanism with the help of a knowledge base.
2. We propose a tree-structure reasoning model based on modular network structure, which has four notable advantages: First, the modular network effectively mines and merges features of different modalities and implements parallel reasoning over tree nodes for logical reasoning. Second, the knowledge base provides some common knowledge other than images and texts to facilitate collaboration and communication between child nodes and parent nodes. Third, the attention mechanism has good interpretability of clearly performing explicit logical reasoning, and the gated reasoning model can tailor and update features.
3. The proposed model has achieved excellent results on benchmark datasets, including VQA2.0, CLVER and FVQA, which indicates that our model has strong adaptability and versatility for different tasks. Through ablative experiments, it can be seen that the contribution of each module network to improving the overall performance of the model.

The structure of the paper is organized as follows: In Section 1, the motivation and outcome of our work are introduced. In Section 2, we present the previous work strongly related to our work and analyze their pros and cons. In Section 3, we show the internal structure and parameters of our model in detail. In Section 4, we conduct experimental analysis and visual reasoning on different datasets. In Section 5, we present the conclusions and expectations of our work.

## 2. Related Work

### 2.1. Visual Question Answering

Visual question answering (VQA) is an intersecting topic that has emerged in the field of computer vision and natural language processing in recent years. Given the image–question pair, the VQA model needs to infer the correct answer in natural language according to the visual information provided by the image, which requires in-depth mining of the characteristics of the image and the question itself, and a full understanding of the fusion features of the image and text. Previous common VQA solutions used CNN-RNN-based [15,16] architectures trained in an end-to-end method, which randomly fused linguistic embedding and visual semantics as clues for predicting the answer. In order to obtain high-dimensional features and reduce the amount of high-complexity computation, bilinear pooling methods [17,18] exploited the fusion of multi-modal features in a fine-grained state. Although these methods introduce the joint embedding of the whole image and text representation, they also introduce redundant noise, which affected the accuracy of the model to a certain extent. To alleviate this discrepancy, attention mechanisms [19,20,21,22] are widely adopted in the field of visual question answering, which aims to find out the local visual area related to the question on the image. Anderson et al. [23] created a bottom-up and top-down attention mechanism that extracted the candidate regions of the image by Faster-RCNN, that is, the image-guided attention mechanism, and then took advantage of the question-guided attention mechanism to predict the correct answer. However, this method lacks the mining of spatial features of the image and cannot make effective inferences in the process. Santoro et al. [24] designed an RN module for relational reasoning, which can be embedded in any neural network that needs to deal with tasks related to relation inference. Wu et al. [25] proposed a multi-step and hierarchical attention mechanism for reasoning, passing the current attention matrix to the next step to generate new compound objects for inferring correct answers.

Most of the above reasoning models focus on images and questions, ignoring the external knowledge base in the field of natural language processing. Moreover, the optimal reasoning structure can adapt to different types of questions, so it is appropriate to have inference structures that are specific to the input question. According to the structure of the text itself, our model uses a neural modular network and attention mechanism for visual reasoning

### 2.2. Knowledge Base

A knowledge base (KB) stores complex structured fact entries in some hierarchical type such as tuple form (S,R,T), where S means subjects, R means the relationship between S and T, and T means targets. The application of knowledge base in visual question answering has attracted increasing attention. Wang et al. [26] proposed a fact-based VQA model which answered the questions by extracting supporting facts from the knowledge base which is a subset of three structured datasets: DBPedia, WebChild, and ConceptNet, and learning the query mappings. However, in the reasoning stage, the relationship between image content and knowledge-based facts is not considered for reasoning. Wu et al. [27] introduced the AMA model of combining the automatically generated image captions with external knowledge-based facts and attribute vectors for predicting the answers. The disadvantage of the model is that it is not interpretable and cannot be used for explicit reasoning based on image features. To bridge this discrepancy, Wang et al. [28] presented the Ahab model to detect the relevant visual content of the image and associate it with the knowledge base and then the question was converted to a query through multiple layers of reasoning for obtaining the final answer. This approach does not have extensive applicability. Once the particular type of question exceeds the scope of the question templates, the accuracy of the model decreases. Yu et al. [29] formulated knowledge-based visual question answering as a recurrent reasoning process for obtaining complementary evidence from multimodal information. Marino et al. [30] addressed the task of knowledge-based visual question answering and provided a benchmark where the image features relied on external knowledge resources. Different from the above methods, our model deeply combines the supporting facts from the knowledge base with image features, and utilizes the fused features for reasoning and answering questions.

### 2.3. Neural Module Network

Neural module networks (NMN) play a significant role in generating layouts that constructs deep networks with flexible computational expressions for addressing compositional visual reasoning. Instead of adopting a fixed network structure, this method assembles a task-specific network from a set of predefined sub-models according to various tasks. Hu et al. [8] proposed using sequence-to-sequence GRU to form a layout, which made multi-step reasoning through a neural module network. Andreas et al. [31] presented a neural module network using a fixed layout generated from a dependency parser. Later, the dynamic neural network model [32] learned to assemble modules by optimizing a limited layout from a list of three to ten candidates, and then trained the weights of these modules, so as to form a new structure for reasoning. Our neural module network not only deeply explores the feature of the image and question, but also fuses the knowledge base to enhance the logical reasoning ability of the model.

## 3. Approach

**Figure 2:**
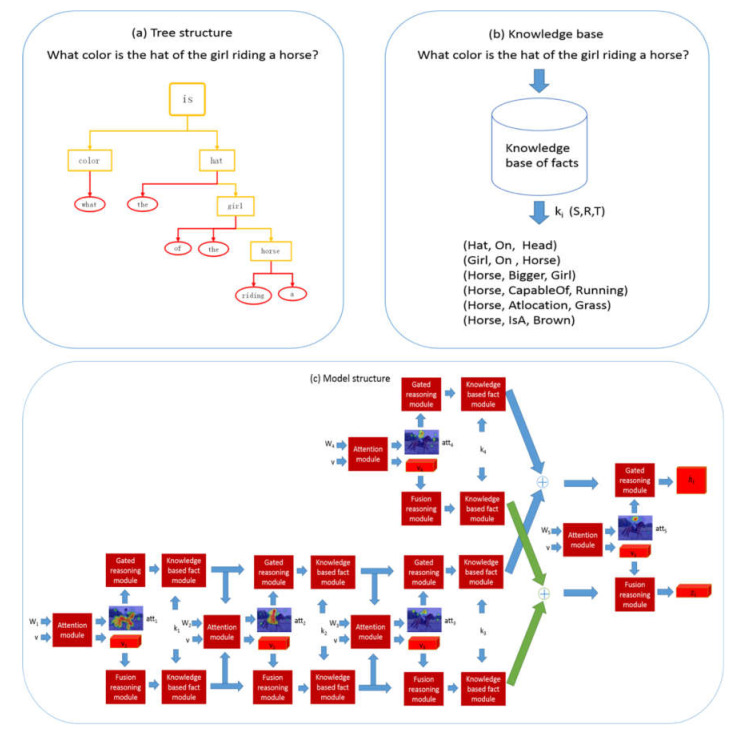
The framework of the proposed QGTSKB model. (**a**) The tree-structured layout is generated by parsing the question with the Stanford Parser. (**b**) The model queries the common sense of each noun in the question from the knowledge base. (**c**) Based on the four neural module networks, our model follows the bottom-up direction of the syntax tree for visual reasoning.

We propose a model of a question-guided tree structure with a knowledge base (QGTSKB) for explicit reasoning in visual question answering tasks. The overall structure of the proposed model is shown in Figure 2. It shows the three steps of model operation: parsing the question into a tree structure, extracting supporting facts from the knowledge base, and assembling the whole network using a neural module network.

We introduce model structures in detail in the following four sections. The overview (Section 3.1) describes the entire pipeline of the model. The attention model (Section 3.2) introduces the attention mechanism that locates key regions based on the image features and question embeddings. The reasoning model (Section 3.3) describes the gated reasoning model and fusion reasoning model. The gated reasoning model shows the process of forgetting and updating the features of the attention map *att_i_* and the summed knowledge-based reasoning feature $E_{i}^{h}$. The fusion reasoning model mines the fusion features of the summed knowledge-based reasoning feature $E_{i}^{z}$ and the attended visual feature *v_i_*. The knowledge-based fact model (Section 3.4) introduces external knowledge to make up for the lack of visual and textual information. Answer prediction (Section 3.5) expounds on the loss function and scoring function of the model.

### 3.1. Overview

As shown in Figure 2a, the tree-structured layout is generated by parsing the question with the Stanford Parser [33] which analyzes the structure of sentences and the relationship between syntactic components at each level. We prune words that are not essential for reasoning, such as prepositions (a, an, the), retaining the leaf node of nouns. Too many leaf nodes can easily cause the model to overfit the data, reducing the complexity of the tree by pruning to avoid overfitting. The tree-structured layout is represented as a 4-tuple *L = (v, q, T, K)*, where *v* represents visual features, *q* represents question embedding, *T* denotes the nodes in the tree structure, which contains the word embedding *w_i(i=_*_1*:N)*_ from the question, and *K* denotes knowledge-based supporting facts in the form *K = (S, R, T)*.

As shown in Figure 2b, the model queries the common sense of each noun in the question from the knowledge base, then the knowledge mapping is formed by the candidates.

As shown in Figure 2c, our model follows the bottom-up direction of the syntax tree for visual reasoning. The image features *v* are extracted from the conv4 features of the ResNet-101 [34]. The question is split into consecutive words, which are encoded by the glove embedding algorithm of a look-up table with 300-dimensional vectors. Furthermore, the fixed-length word embedding *w* is generated by the hidden vector of bi-directional gated recurrent unit (Bi-GRU) in the original order. The question embedding *q* is denoted by the hidden vector from the last hidden state of Bi-GRU.

The neural module network composes a model with logical reasoning ability according to the layout of the tree structure. The input of the model includes an image feature *v*, a question embedding *q*, a word embedding *w_i_* and supporting knowledge-based facts *k*. The attention module *f_a_* creates the attention map *att_i_* based on the image feature v, wording embedding *w_i_* and the summed knowledge-based reasoning feature $E_{i}^{h}$ and $E_{i}^{z}$. The gated reasoning model *f_g_* takes advantage of the structure of the Gated Recurrent Unit to forget and update the attention map *att_i_*. The fusion reasoning model *f_z_* deeply mines high-level semantics of the attended visual feature *v_i_* and knowledge base. The knowledge-based fact model provides the external knowledge for reasoning.

### 3.2. Attention Model

**Figure 3:**
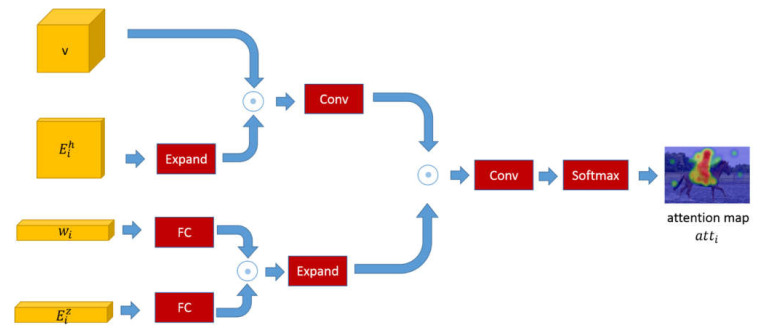
The internal structure of the attention model. The model focuses on the attended image features upon image features and word embeddings.

In leaf node *i*, the attention module *f_a_* generates an attention map over the whole image, which is used to locate key objects or attributes related to the image features and the wording embedding. As indicated in Figure 3, to ensure that the image feature *v* and the summed knowledge-based reasoning feature $E_{i}^{h}$ have the same dimension, we expand the matrix $E_{i}^{h}$ to fit the image feature matrix *v*. *c_v_* is produced by the element-wise multiplication of matrix $E_{i}^{h}$ and matrix *v*. The word embedding *w_i_* and the summed knowledge-based reasoning feature $E_{i}^{z}$ are passed through a fully connected layer to perform spatial mapping transformation and then made in an element-wise multiplication operation. We map *c_v_* and *c_w_* to 2048-dimensional features and conduct element-wise multiplication on these two products. The result passes through a convolutional layer and a softmax layer for regularizing the final attention map into the range (0, 1). For visual attentions, the attended visual feature *v_i_* of the node *i* is represented by the weights of attention maps *att_i_* multiplied by the image feature *v* as:

(1) $$c_{v}=v⊙E_{i}^{h}$$

(2) $$c_{w}=(w_{1}w_{i})⊙(w_{2}E_{i}^{z})$$

(3) $$att_{i}=\mathrm{softmax}(\mathrm{ReLU}(w_{3}(c_{v}⊙c_{w})))$$

(4) $$v_{i}=\sum_{i=1}^{N}att_{i}\cdot v$$

where *w*_1_, *w*_2_, *w*_3_ are learned parameters, and ⊙ denotes the element-wise multiplication.

### 3.3. Reasoning Model

**Figure 4:**
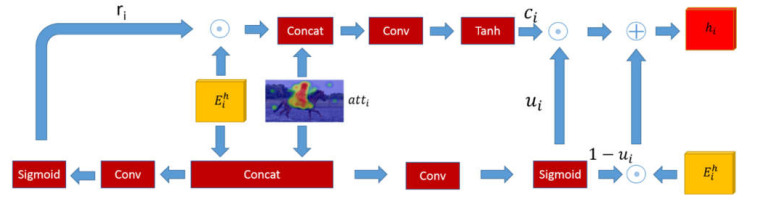
The framework of the gated reasoning model. The gated reasoning model forgets and updates the features of the attention map atti and the summed knowledge-based reasoning feature $E_{i}^{h}$.

In this section, we introduce the internal structure of the gated reasoning model and fusion reasoning model. As shown in Figure 4, the gated reasoning model takes advantage of the structure of the Gated Recurrent Unit which is a simplified version of the LSTM with fewer parameters. It adopts a gated mechanism to get rid of the problem of gradient vanishing and gradient explosion. The input of the gated reasoning model is the summed knowledge-based reasoning feature $E_{i}^{h}$ and attention map *att_i_*, and output of the model is the hidden representation of the final state *h_i_*. The model has two kinds of gated units which are reset gate and update gate. Intuitively, the reset gate determines how to combine the feature $E_{i}^{h}$ with the attention map *att_i_*. The concatenation of these two features passes through a convolutional layer to perform feature fusion and then through a sigmoid layer to update a reset matrix *r_i_*. The function of the update gate is to control the amount of information flow, that is, to select valuable parts of the feature $E_{i}^{h}$ and the attention map *att_i_* saved into the current state. The calculation method of gated threshold *u_i_* is the same as the process of calculating reset value *r_i_*. The element-wise product of the reset *r_i_* and the feature $E_{i}^{h}$ is saved in the memory cell *c_i_.* The gated threshold *u*_i_ is created by the element-wise multiplication instead of simple multiplication for adjusting the amount of the memory cell *c_i_* and the feature $E_{i}^{h}$ retained in the hidden representation of the final state *h_i_* as:

(5) $$E_{i}^{h}=\sum_{i\in C}e_{i}^{h}$$

(6) $$u_{i}=\sigma (w_{u}\cdot [E_{i}^{h},att_{i}])$$

(7) $$r_{i}=\sigma (w_{r}\cdot [E_{i}^{h},att_{i}])$$

(8) $$c_{i}=\tanh(w_{c}\cdot [r_{i}⊙E_{i}^{h},att_{i}])$$

(9) $$h_{i}=(1-u_{i})⊙E_{i}^{h}+u_{i}⊙c_{i}$$

where *w_u_*, *w_r_* are learned parameters, and [ , ] denotes concatenation operation.

**Figure 5:**
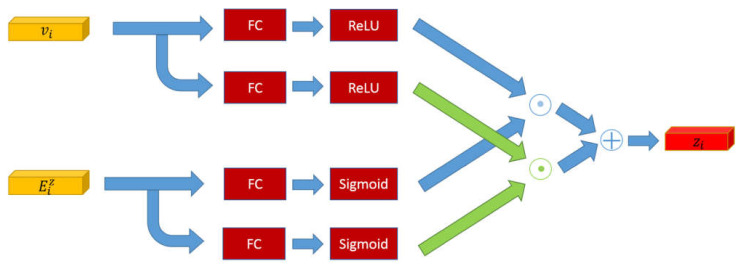
The structure of the fusion reasoning model. It mines high-level semantics of the attended visual features and knowledge-based facts.

As shown in Figure 5, the fusion model *f_z_* generates fusion reasoning features *z_i_* based on the attended visual features *v_i_* and the summed knowledge-based reasoning feature $E_{i}^{z}$. Low-level feature fusion concatenates original features to create a feature vector. However, it has a small effect on improving the reasoning ability of the model. The fusion operation increases the change of features and obtains higher-level semantic features. A fully connected layer changes the spatial structure of features. The sigmoid and ReLU layer act as the activation functions to enhance the nonlinear mapping of the model as:

(10) $$E_{i}^{z}=\sum_{i\in C}e_{i}^{z}$$

(11) $$z_{i}=[\mathrm{ReLU}(w_{4}v_{i})⊙\sigma (w_{5}E_{i}^{z})]⊕[\mathrm{ReLU}(w_{6}v_{i})⊙\sigma (w_{7}E_{i}^{z})]$$

where *w*_4_, *w*_5_, *w*_6_, *w*_7_ are learned parameters and ⊕ denotes the element-wise summation.

### 3.4. Knowledge-Based Fact Model

The knowledge base compensates for the shortage of features of images and texts by providing facts related to the entities in the question. In the leaf node *i*, *k_i_* is the concatenation of supporting facts and the question. The supporting fact *k_i_* (*S, R, T*) can be obtained by extracting the entity in the question and querying the entity in the knowledge base. For example, given the question ”What color is the hat of the girl riding a horse?”, the supporting fact (Hat, on, Head) indicates the spatial relationship between the hat and the girl and the supporting fact (Horse, bigger, Girl) presents the size difference between a girl and a horse. The supporting facts provide sufficient clues to locate the related objects for answering questions and logical reasoning.

**Figure 6:**
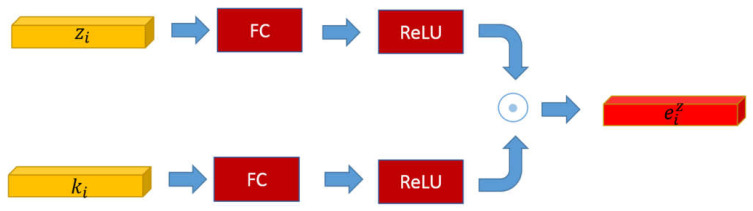
The framework of the knowledge-based fact model. The knowledge-based reasoning feature $e_{i}^{z}$ is generated by the element-wise multiplication of the fusion reasoning feature *z_i_* and the knowledge-based supporting fact *k_i_*.

As shown in Figure 6, the knowledge-based supporting fact *k_i_* and the fusion reasoning feature *z_i_* go through the fully connected layer and the activation layer (ReLU) which performs spatial transformation and obtains the same dimension, respectively. The knowledge-based reasoning feature $e_{i}^{z}$ is achieved by the element-wise multiplication of these two resulting vectors as:

(12) $$e_{i}^{z}=[\mathrm{ReLU}(w_{8}z_{i})]⊙[\mathrm{ReLU}(w_{9}k_{i})]$$

where *w*_8_, *w*_9_ are learned parameters.

### 3.5. Answer Prediction

Traversing each node of the tree from bottom to top, our model obtains the final result $e_{i}^{z}$ and $e_{i}^{h}$ at the root of the tree. The result $e_{i}^{h}$ passes through a convolutional layer and concatenates with the vector $e_{i}^{z}$. The concatenated vector is fed into a softmax layer to achieve the probability s as the answers for the question–image pair. Formally,

(13) $$s=\mathrm{softmax}(\mathrm{ReLU}(w_{11}[w_{10}e_{i}^{h},e_{i}^{z}]))$$

where *w*_10_, *w*_11_ are learned parameters.

Since there is only one candidate labeled as the ground-truth answer and the rest of the candidates are annotated as negative answers in each training batch, it is efficient to adopt binary cross-entropy loss [35] to deal with the question–image pairs as:

(14) $$L=\underset{i}{\sum^{M}}\;\underset{j}{\sum^{N}}\;\left(1-s_{i}^{j}\right)\log\left(1-{\hat{s}}_{i}^{j}\right)-s_{i}^{j}\log\left({\hat{s}}_{i}^{j}\right)$$

where *M* represents the batch size and *N* represents the number of candidate answers. $s_{i}^{j}$ is the ground truth label while ${\hat{s}}_{i}^{j}$ is the predicted probability.

## 4. Experiments

### 4.1. Datasets

CLVER [36] is a synthesized dataset including a training set, a validation set and a test set. It contains images of 3D-rendered objects of different sizes, shapes, material types, and colors. Each question in CLEVR is generated by a functional program in natural language. The questions in the data set always require a series of reasoning processes. In order to evaluate the reasoning ability in detail, all questions are divided into 5 categories: Exist, Count, Compare Integer, Query Attribute and Compare Attribute.

The VQA v2.0 [37] is a widely used dataset containing open-ended questions about images. It is an updated version of the VQA v1.0, which has less data bias. It contains 265,016 images from COCO [38], more than 3 questions per image, 10 ground-truth answers per question and 3 plausible answers per question. There are three types of answers: yes/no, number and other.

The FVQA dataset contains 193,499 facts corresponding to the questions. The knowledge base is constructed by querying top visual concepts from three knowledge bases: DBPedia, ConceptNet and WebChild.

### 4.2. Implementation Details

In our experiment on the CLVER dataset, each image is resized to 224 × 224. Furthermore, we extract the 1024 × 14 × 14 feature maps by forwarding the 3 × 224 × 224 resized image through the res4b22 layer of the Resnet-101 with the kernel size of (1, 1) and (3, 3) trained on the ImageNet classification. For improving spatial reasoning, we connect two coordinates $\frac{x}{14}$ and $\frac{y}{14}$ with the resulting feature map, which passes through a convolution layer to obtain a 128 × 14 × 14 feature map. For question and supporting facts representations, each word of the question and supporting fact is embedded by 300-dimensional GloVe word embeddings [39]. The sequence of the word embeddings is fed into a bidirectional GRU with a 512-dimensional hidden layer for both directions. Each word embedding is extracted from the hidden vector at the corresponding position of the Bi-GRU. The output of the knowledge-based model and gated reasoning module are 128 × 14 × 14 for both hidden representation $h_{i}$ and knowledge-based features $E_{i}^{h}$. The attended visual feature and the feature $v_{i}$ are 128 dimensions.

During supervised training, our model is trained by Adam optimizer [40] with 12 epochs, where the batch size is 128 and 64 for the VQA v2.0 and CLVER dataset, respectively. The learning rate is 0.0003 and 0.0001 for CLVER and VQA v2.0, respectively. The $\beta_{1}$ is 0.9 and $\beta_{2}$ is 0.999. The model is trained on the training datasets and evaluated on the test or validation dataset.

### 4.3. Comparison with Existing Methods

We evaluate our model and the previous model on each question type in the CLVER dataset. As shown in Table 1, the Q-type model of predicting the most frequent answer based on a question’s category obtains the lowest score. In the LSTM model, questions are encoded by the word embedding and then are fed into LSTM. The result is passed to an MLP layer to predict a probability distribution of answers, which has no obvious improvement compared to the previous one. Compared with the first two models, the accuracy of the CNN + LSTM model performing reasoning based on the image features extracted by the CNN and question features encoded by the LSTM is slightly improved. “N2NMN scratch” using reinforcement learning with layout supervision has a good performance in the Query Attribute dataset. “N2NMN cloning expert” utilizes the supervised layout from the expert policy. Compared with the “N2NMN scratch”, the accuracy of the model is greatly improved by 10%, which indicates that full supervision helps improve the model’s logical reasoning ability. “N2NMN policy search” achieves higher performance in all aspects by further training the parser from “N2NMN cloning expert”. “PG + EE (9 K prog.)” and “PG + EE (700 K prog.)” use 9 K and 700 K ground-truth programs, which indicates the model has a deeper understanding of features as the number of ground-truth programs increases. Our model outperforms the previous methods without using a fixed layout. Compared with the RN model, our model has a higher accuracy rate and explicit reasoning. The knowledge base helps our model to effectively reason about complex questions.

We compare our model with previous methods on the VQA v2.0 in Table 2. The accuracy of our model is greatly improved, which is 2% higher than that of the second place. It gains obvious progress in answering “Yes/No” questions. Compared with the above algorithms, our model not only has certain advantages in accuracy, but also has the function of logical reasoning. The multiple glimpse, bidirectional attention, stacked attention and memory unit method are used in these methods. However, these models are like black boxes and cannot make explicit visual inferences based on the content of the question. Moreover, compared with the above models, our model introduces a knowledge base. The knowledge-based supporting facts are effectively fused with image features and question features to perform modular reasoning.

### 4.4. Visual Reasoning

**Figure 7:**
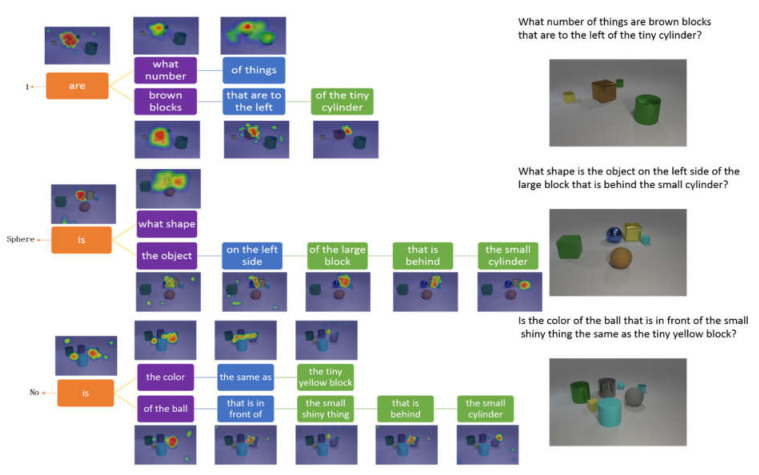
Three exanples of the model performing visual reasoning by focusing on related regions according to the words on CLVER.

In this section, we introduce the process of visual reasoning based on the tree structure parsed from the question. The CLVER dataset is generated by codes for reasoning. Thus, the knowledge base provides limited supporting facts for the nouns in the question. As shown in Figure 7, the first example shows that our model counts the number of brown blocks. The first step is to find the position of the tiny cylinder, and secondly, our model defines spatial location relationship according to the “left”. The third step is to locate the brown square and count its number. Finally, the root node extracts the fused feature and predicts the answer “1”. The second example indicates that our model can figure out the shape of objects. Firstly, our model locates the small cylinder and then searches out the “behind” spatial relationship. Later, it finds out the large block based on the fused features and searches for the “left” spatial relationship. Finally, the root node “is” predicts the answer “Sphere”. The third example shows that our model can identify the color of an object through complex reasoning. The model locates objects and finds out the spatial position relationship according to the question multiple times. The fused feature of “ball” and “color” is passed through the root node “is” to obtain the probability distribution of the question. These examples prove that our model can be adapted to many types of tasks, and the attention mechanism clearly shows the reasoning process of the model. The prominent feature of the model is the flexibility to adjust network structures according to the structure of the question.

**Figure 8:**
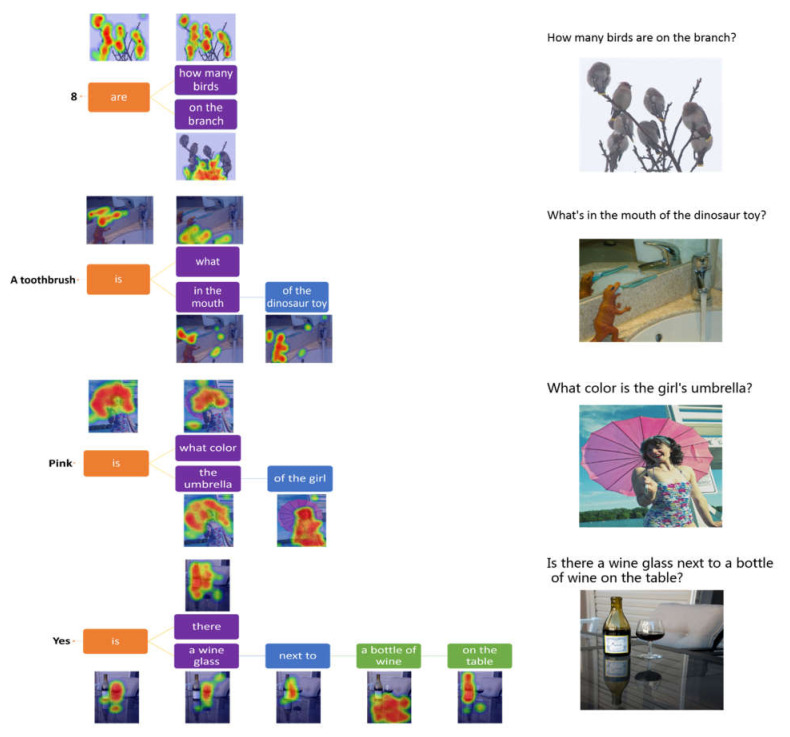
Examples of the model performing visual reasoning by focusing on related regions according to the words on VQA v2.0.

We also evaluate the interpretability of the model on the VQA v2.0 dataset which contains open-ended questions and realistic images. Questions require an understanding of features of images, questions and commonsense knowledge to answer. As shown in Figure 8, the first example shows that the model counts out the number of birds on the branch based on the supporting knowledge facts. The second example indicates that the model can identify the object in the dinosaur’s mouth. The third example demonstrates that our model can identify the color of the object based on the fused feature of the umbrella and the girl. The fourth example shows that our model can deal with complex spatial relations between the objects. Through the above four examples, it can be concluded that our model has excellent adaptability, stability and interpretability for different question types.

## 5. Conclusions

In this paper, we propose a reasoning model of a question-guided tree structure with a knowledge base (QGTSKB) for visual question answering. To the best of our knowledge, this is the first visual reasoning model that leverages the combination of the entire language structure, visual features and knowledge base. Our model can automatically perform interpretable visual reasoning over a parsing tree structure from the question, which has strong flexibility and universality of adjusting the structure according to different types of questions. Compared with previous methods, it does not rely on annotations or manual rules to set the network layout. The knowledge base provides supporting facts making up for the lack of the original visual features and question features for reasoning. The neural modular network simplifies a single large-scale into small and manageable modules. Each module of the neural module network has specific functions and is independent of other modules. Due to parameter sharing, a modular neural network reduces computation cost and improves the efficiency of the model. Our model obtains excellent results and performs visual logical reasoning on the VQA v2.0 and CLVER dataset.

Our model uses convolutional neural networks to extract visual features of objects as representations of images. However, it cannot accurately capture the relationship between objects. We are going to adopt the method of graph network to model the fully connected graph according to the relationship of objects in the image, in which the objects in the image are represented as nodes of the image and the relationship between objects is represented as edges of the image. How to deeply mine object relationships in images to establish a graph neural network for reasoning on visual question answering is essential.

## Figures and Tables

**Table 1:** Accuracy comparison between the proposed method and the existing methods on CLVER dataset.

Model	Overall	Count	Exist	Compare Numbers	Query Attribute	Compare Attribute
Q-type model [36]	41.8	34.6	50.2	51.0	36.0	51.3
LSTM [36]	46.8	41.7	61.1	69.8	36.8	51.8
CNN + LSTM [36]	52.3	43.7	65.2	67.1	49.3	53.0
N2NMN scratch [8]	69.0	55.1	72.7	78.5	83.2	50.9
N2NMN cloning Expert [8]	78.9	63.3	83.3	80.3	87.0	78.5
N2NMN policy search [8]	83.7	68.5	85.7	84.9	90.0	88.7
PG + EE(9 K prog.) [7]	88.6	79.7	89.7	79.1	92.6	96.0
PG + EE(700 K prog.) [7]	96.9	92.7	97.1	98.7	98.1	98.9
RN [24]	95.5	90.1	97.8	93.6	97.9	97.1
QGTSKB (Ours)	97.6	94.2	99.1	93.6	99.3	99.2

**Table 2:** Performance comparison on VQA v2.0 dataset.

Model	Test-Dev				Test-Standard
	Overall	Yes/No	Number	Other	Overall
QTA [4]	57.99	80.87	37.32	43.12	58.24
SAN [22]	58.7	79.3	36.6	46.1	58.9
HQIC [3]	61.8	79.7	38.7	51.7	62.1
DAN [5]	64.3	83	39.1	53.9	64.2
MFB [17]	65.9	84	39.8	56.2	65.8
DCN [21]	66.89	84.61	42.35	57.31	67.02
Count [16]	68.09	83.14	51.62	58.97	68.41
QGTSKB (Ours)	70.5	86.76	52.54	60.68	70.62

## Data Availability

Publicly available datasets were analyzed in this study. Available online: https://visualqa.org/download.html (accessed on 26 August 2021).

## References

[1] Shih K.J., Singh S., Hoiem D. Where To Look: Focus Regions for Visual Question Answering. Proceedings of the 2016 IEEE Conference on Computer Vision and Pattern Recognition (CVPR).

[2] Fukui A., Park D.H., Yang D., Rohrbach A., Darrell T., Rohrbach M. (2016). Multimodal compact bilinear pooling for visual question answering and visual grounding. Proceedings of the 2016 Conference on Empirical Methods in Natural Language Processing, EMNLP 2016.

[3] Lu J., Yang J., Batra D., Parikh D. Hierarchical Question-Image Co-Attention for Visual Question Answering. Proceedings of the 30th Conference on Neural Information Processing Systems (NIPS).

[4] Shi Y., Furlanello T., Zha S., Anandkumar A. Question Type Guided Attention in Visual Question Answering. Proceedings of the European Conference on Computer Vision (ECCV).

[5] Nam H., Ha J.-W., Kim J. (2017). Dual attention networks for multimodal reasoning and matching. Proceedings of the 30th IEEE Conference on Computer Vision and Pattern Recognition, CVPR 2017.

[6] Manjunatha V., Saini N., Davis L.S., Soc I.C. Explicit Bias Discovery in Visual Question Answering Models. Proceedings of the 32nd IEEE/CVF Conference on Computer Vision and Pattern Recognition (CVPR).

[7] Johnson J., Hariharan B., Van Der Maaten L., Hoffman J., Fei-Fei L., Lawrence Zitnick C., Girshick R. Inferring and Executing Programs for Visual Reasoning. Proceedings of the 16th IEEE International Conference on Computer Vision (ICCV).

[8] Hu R., Andreas J., Rohrbach M., Darrell T., Saenko K. (2017). Learning to Reason: End-to-End Module Networks for Visual Question Answering. Proceedings of the 16th IEEE International Conference on Computer Vision, ICCV 2017.

[9] Zhang W.F., Yu J., Hu H., Hu H.Y., Qin Z.C. (2020). Multimodal feature fusion by relational reasoning and attention for visual question answering. Inf. Fusion.

[10] Cao Q., Liang X., Li B., Lin L. (2021). Interpretable Visual Question Answering by Reasoning on Dependency Trees. IEEE Trans. Pattern Anal. Mach. Intell..

[11] Cao Q., Liang X., Li B., Li G., Lin L. (2018). Visual Question Reasoning on General Dependency Tree. Proceedings of the 31st Meeting of the IEEE/CVF Conference on Computer Vision and Pattern Recognition, CVPR 2018.

[12] Auer S., Bizer C., Kobilarov G., Lehmann J., Cyganiak R., Ives Z. (2007). DBpedia: A nucleus for a Web of open data. Proceedings of the 6th International Semantic Web Conference, ISWC 2007 and 2nd Asian Semantic Web Conference, ASWC 2007.

[13] Tandon N., De Melo G., Suchanek F., Weikum G. (2014). WebChild: Harvesting and organizing commonsense knowledge from the web. Proceedings of the 7th ACM International Conference on Web Search and Data Mining, WSDM 2014.

[14] Speer R., Chin J., Havasi C. ConceptNet 5.5: An Open Multilingual Graph of General Knowledge. Proceedings of the 31st AAAI Conference on Artificial Intelligence.

[15] Ren M., Kiros R., Zemel R.S. (2015). Exploring models and data for image question answering. Proceedings of the 29th Annual Conference on Neural Information Processing Systems, NIPS 2015.

[16] Zhang Y., Hare J., Prugel-Bennett A. Learning to count objects in natural images for visual question answering. Proceedings of the 6th International Conference on Learning Representations, ICLR 2018.

[17] Yu Z., Yu J., Fan J., Tao D. Multi-modal Factorized Bilinear Pooling with Co-Attention Learning for Visual Question Answering. Proceedings of the 16th IEEE International Conference on Computer Vision (ICCV).

[18] Ben-Younes H., Cadene R., Thome N., Cord M. (2019). BLOCK: Bilinear superdiagonal fusion for visual question answering and visual relationship detection. Proceedings of the 33rd AAAI Conference on Artificial Intelligence, AAAI 2019, 31st Annual Conference on Innovative Applications of Artificial Intelligence, IAAI 2019 and the 9th AAAI Symposium on Educational Advances in Artificial Intelligence, EAAI 2019.

[19] Xu H., Saenko K. (2016). Ask, attend and answer: Exploring question-guided spatial attention for visual question answering. Proceedings of the 21st ACM Conference on Computer and Communications Security, CCS 2014.

[20] Zhu Y., Groth O., Bernstein M., Fei-Fei L. (2016). Visual7W: Grounded question answering in images. Proceedings of the 29th IEEE Conference on Computer Vision and Pattern Recognition, CVPR 2016.

[21] Nguyen D.-K., Okatani T. (2018). Improved Fusion of Visual and Language Representations by Dense Symmetric Co-attention for Visual Question Answering. Proceedings of the 31st Meeting of the IEEE/CVF Conference on Computer Vision and Pattern Recognition, CVPR 2018.

[22] Yang Z., He X., Gao J., Deng L., Smola A. (2016). Stacked attention networks for image question answering. Proceedings of the 29th IEEE Conference on Computer Vision and Pattern Recognition, CVPR 2016.

[23] Anderson P., He X., Buehler C., Teney D., Johnson M., Gould S., Zhang L. (2018). Bottom-Up and Top-Down Attention for Image Captioning and Visual Question Answering. Proceedings of the 31st Meeting of the IEEE/CVF Conference on Computer Vision and Pattern Recognition, CVPR 2018.

[24] Santoro A., Raposo D., Barrett D.G., Malinowski M., Pascanu R., Battaglia P., Lillicrap T. (2017). A simple neural network module for relational reasoning. Proceedings of the 31st Annual Conference on Neural Information Processing Systems, NIPS 2017.

[25] Wu C., Liu J., Wang X., Dong X. (2018). Chain of reasoning for visual question answering. Proceedings of the 32nd Conference on Neural Information Processing Systems, NeurIPS 2018.

[26] Wang P., Wu Q., Shen C., Dick A., van den Hengel A. (2018). FVQA: Fact-Based Visual Question Answering. IEEE Trans. Pattern Anal. Mach. Intell..

[27] Wu Q., Wang P., Shen C., Dick A., van den Hengel A. (2016). Ask me anything: Free-form visual question answering based on knowledge from external sources. Proceedings of the 29th IEEE Conference on Computer Vision and Pattern Recognition, CVPR 2016.

[28] Wang P., Wu Q., Shen C., Dick A., van den Hengel A. Explicit knowledge-based reasoning for visual question answering. Proceedings of the 26th International Joint Conference on Artificial Intelligence, IJCAI 2017.

[29] Yu J., Zhu Z., Wang Y., Zhang W., Hu Y., Tan J. (2020). Cross-modal knowledge reasoning for knowledge-based visual question answering. Pattern Recognit..

[30] Marino K., Rastegari M., Farhadi A., Mottaghi R. OK-VQA: A Visual Question Answering Benchmark Requiring External Knowledge. Proceedings of the 2019 IEEE/CVF Conference on Computer Vision and Pattern Recognition (CVPR).

[31] Andreas J., Rohrbach M., Darrell T., Klein D. (2016). Neural module networks. Proceedings of the 29th IEEE Conference on Computer Vision and Pattern Recognition, CVPR 2016.

[32] Andreas J., Rohrbach M., Darrell T., Klein D. (2016). Learning to compose neural networks for question answering. Proceedings of the 15th Conference of the North American Chapter of the Association for Computational Linguistics: Human Language Technologies, NAACL HLT 2016.

[33] Chen D., Manning C.D. (2014). A fast and accurate dependency parser using neural networks. Proceedings of the 2014 Conference on Empirical Methods in Natural Language Processing, EMNLP 2014.

[34] He K., Zhang X., Ren S., Sun J. (2016). Deep residual learning for image recognition. Proceedings of the 29th IEEE Conference on Computer Vision and Pattern Recognition, CVPR 2016.

[35] Ruby U., Yendapalli V. (2020). Binary cross entropy with deep learning technique for Image classification. Int. J. Adv. Trends Comput. Sci. Eng..

[36] Johnson J., Fei-Fei L., Hariharan B., Zitnick C.L., van der Maaten L., Girshick R. (2017). CLEVR: A diagnostic dataset for compositional language and elementary visual reasoning. Proceedings of the 30th IEEE Conference on Computer Vision and Pattern Recognition, CVPR 2017.

[37] Goyal Y., Khot T., Agrawal A., Summers-Stay D., Batra D., Parikh D. (2019). Making the V in VQA Matter: Elevating the Role of Image Understanding in Visual Question Answering. Int. J. Comput. Vis..

[38] Lin T.Y., Maire M., Belongie S., Hays J., Perona P., Ramanan D., Dollár P., Zitnick C.L. (2014). Microsoft COCO: Common objects in context. Proceedings of the 13th European Conference on Computer Vision, ECCV 2014.

[39] Pennington J., Socher R., Manning C.D. (2014). GloVe: Global vectors for word representation. Proceedings of the 2014 Conference on Empirical Methods in Natural Language Processing, EMNLP 2014.

[40] Kingma D.P., Ba J.L. Adam: A method for stochastic optimization. Proceedings of the 3rd International Conference on Learning Representations, ICLR 2015, International Conference on Learning Representations, ICLR.

